# Topical Film-Forming Chlorhexidine Gluconate Sprays for Antiseptic Application

**DOI:** 10.3390/pharmaceutics14061124

**Published:** 2022-05-25

**Authors:** Benchawan Chamsai, Sirima Soodvilai, Praneet Opanasopit, Wipada Samprasit

**Affiliations:** 1 Department of Pharmaceutical Technology, College of Pharmacy, Rangsit University, Pathum Thani 12000, Thailand; benchawan.c@rsu.ac.th (B.C.); sirima.s@rsu.ac.th (S.S.); 2 Department of Pharmaceutical Technology, Faculty of Pharmacy, Silpakorn University, Nakhon Pathom 73000, Thailand; opanasopit_p@su.ac.th

**Keywords:** film-forming sprays, chlorhexidine gluconate, antiseptic, topical delivery

## Abstract

Topical film-forming sprays of chlorhexidine gluconate (CHG-FFS) were developed for antiseptic application. Various polymers and solvents were studied for their potential as film-forming polymers and solvent systems, respectively. To produce CHG-FFS, the optimal polymer and solvent were selected, and their physicochemical properties were evaluated. The in vivo evaluation of CHG-FFS was investigated for the satisfaction of the dosage forms, time required for the film formation, film appearance, and adhesion on the skin. Antibacterial activity was also studied in vitro and in vivo. The optimized formulation was assessed for the in vitro cell line evaluations of the cytotoxicity and wound healing. The results demonstrate that Eudragit^®^ S100, Eudragit^®^ L100, and polyvinyl alcohol (PVA) have the ability to be used as film-forming polymers in an ethanolic solution. A clear and flexible film was obtained from transparent homogenous solutions of CHG-FFS after actuation. They generated the fast thin film formation on the skin with the satisfaction of the dosage forms. Furthermore, the formulations inhibited the growth of *Staphylococcus aureus* in vitro and provided antiseptic activity in vivo. However, PVA was found to be an optimal film-forming polymer for promoting CHG adhesion on the skin. The CHG-FFS obtained from the PVA also provided a CHG film, which was non-toxic to human skin cells and did not interfere with the wound healing process. Therefore, the developed CHG-FFS could be a promising candidate for topical antiseptic application.

## 1. Introduction

The skin is a valuable route for drug administration, imparting both local and systemic effects. To improve treatment efficiency, drugs for the local effect are usually formulated in a dose form, such as a cream, lotion, gel, ointment, patch, or spray. However, conventional formulations have shortcomings including poor skin adherence, permeability, and patient compliance. Semi-solid dosage forms also easily attach to clothing while mobile and, because they are applied with the fingers, might promote wound cross-infection [1]. Alternatively, the medicated adhesive patch is applied topically to deliver a specified dose of the drug; however, it still leaves drug residue after use and can be deliberately abused [1]. Film-forming spray (FFS) is a novel approach, which can be used as an alternative for traditional topical and transdermal dosage forms. FFS appears in the form of a sprayed solution which produces a thin film on the skin after being sprayed [2]. Thin film forms can increase the contact time and permeability of the drug that is similar to a patch [1]. Furthermore, FFS can form films following the pattern of the skin or wound, since deep indentations can be exposed to small droplets of the film-forming solution [1].

**Scheme 1:**
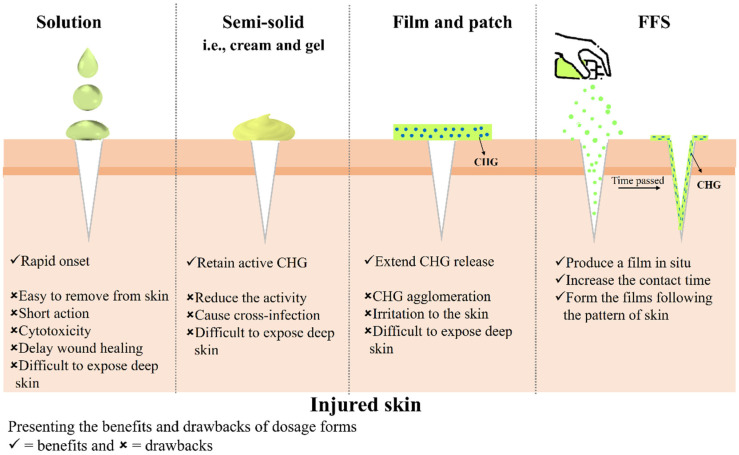
Mechanism of conventional formulations in comparison to the FFS system presenting the film formation, benefits, and drawbacks of each dosage form.

Skin infections are inflammatory microbial infections that affect the skin layers [3]. The most common pathogens associated with skin infection are Gram-positive infections, such as *Staphylococcus aureus* [4]. In order to prevent infections in wounds, skin infections are usually treated with a topical antiseptic agent [5,6]. Antiseptics are also becoming more widely used for hand cleaning and skin decolonization as important strategies for preventing healthcare-associated infections. Chlorhexidine gluconate (CHG) is one of the most frequently used topical antiseptics. It is a bactericidal or bacteriostatic bisbiguanide antiseptic and disinfectant that acts against Gram-positive and Gram-negative bacteria [7]. CHG is commonly observed in lotions, washes, and creams for skin disinfection and wounds, as well as mouthwashes for oral infections and plaque reduction. It is generally provided in a solution range from 0.5 to 4% *w*/*v* for topical skin treatment, such as preoperative skin disinfection and handwashing [3,8]. Previous studies stated that CHG was developed as a solution [9], gel [10], cream [11], film [12], and patch [13]. On the other hand, these formulations exhibited considerable disadvantages. The CHG solution had a rapid onset of antiseptic activity; however, it was easy to remove from the skin and the action was short [9]. Furthermore, these solutions have some drawbacks such as the potential for cytotoxicity in fibroblasts, osteoblasts, and lymphocytes in a time- and dose-dependent manner, which could cause a delay in wound healing [14]. The gel and cream were sufficient to retain active CHG concentrations in the active site [13,15]. However, creams containing an emulsifier decreased the antibacterial effect of CHG [11]. CHG could become inactivated when the cream was formulated with the non-ionic surfactants [3]. In addition, CHG could agglomerate in a film, thus resulting in extended CHG release [12]. CHG and patch irritation were found to be unacceptably strong in in vivo studies [13]. Nonetheless, as compared to FFS, all of these formulations made it more difficult to expose deep skin or wounds, suggesting that FFS could be a novel CHG dosage form to minimize the disadvantages and improve patient compliance. Furthermore, FFS may be less cytotoxic in human skin and less likely to interfere with wound healing. Scheme 1 shows the hypothesis and drawbacks of the conventional formulations in comparison to the FFS system.

The aim of this study was to develop the topical FFS of CHG (CHG-FFS). The hypothesis was that CHG-FFS could generate a thin film for topical antiseptic application without producing cytotoxicity in human skin cells. The possibility of various polymers and solvents as film-forming polymers and solvent systems was investigated. The physicochemical properties of CHG-FFS and their films were evaluated. The satisfaction with CHG-FFS, the time required for film formation, the film appearance, and skin adhesion were also studied in vivo. The optimized CHG-FFS was selected to study the antibacterial activity both in vitro and in vivo, cytotoxicity, and potential for wound healing in cell lines.

## 2. Materials and Methods

### 2.1. Materials

Chlorhexidine gluconate (CHG), sodium alginate (ALG), and polyvinylpyrrolidone K30 (PVP K30) were purchased from Sigma, St. Louis, MO, USA. Polyvinyl alcohol (PVA, degree of polymerization ≈1600, degree of hydrolysis ≈97.5–99.5 mol %, and average MW = 77,000–82,000 g/mol) was purchased from Fluka, Switzerland. Sodium carboxyl methylcellulose (CMC) was received from Rama chem., Thailand. Polymethacrylates including Eudragit^®^ E100 (E), Eudragit^®^ L100 (L), Eudragit^®^ S100 (S), and Eudragit^®^ RS100 (RS) were provided as a gift from Evonik Nutrition and Care GmbH Health Care, Darmstadt, Germany. All of the other chemicals were of an analytical grade.

### 2.2. Screening of the Film-Forming Polymers and Solvent Systems

The film-forming polymers were considered based on their potentiality to create transparent films with no wrinkles after the solvent had evaporated, as well as their solubility in the solvent systems. Thus, both water soluble polymers (ALG, CMC, PVA, and PVP K30) and water insoluble polymers (E, L, S, and RS) were used and considered as film-forming polymers. The solvent casting method was used to prepare polymeric films. A polymeric solution was prepared by dissolving the polymer in a suitable solvent, i.e., water and ethanol (EtOH) at the concentration of 1.5% *w*/*w* (Table 1). The solution was poured onto plastic plates with a 10 cm diameter at the same polymer weight and was then dried to allow the solvent to evaporate in a hot air oven set at 37 °C with an indoor temperature of 34–35 °C for 6 h. The solution of the film-forming agents and the dried films were visually inspected for the appearance. The film-forming polymers that provided the clear solution and transparent film were further considered as the suitable film-forming polymers to find the optimized type and concentration of the solvent systems. The organic solvents of EtOH and isopropyl alcohol (IPA) were investigated as solvent systems for the FFS. Several concentrations of EtOH and IPA were prepared as the solvent systems (Table 2). The film-forming polymers’ solubility in the solvents was determined by dissolving a certain fixed amount of the polymer into the solvent systems. The solution of film-forming polymers was examined for the evaporation rate by spraying the solution onto a clean Petri dish and then accurately weighted using an analytical balance to four decimal places. The evaporation rate of each of the various solutions was determined by examining the weight changes over time. The tests were carried out at a room temperature of 25–28 °C and the weights were recorded every 30 s until 10 min. These conditions differed from the physiological temperature of the skin because the room temperature could not be controlled to 35 °C. However, the solvent behavior to evaporate and the evaporation rate could be observed when the solution was applied at an ambient temperature. The tests were repeated three times, and graphs of weight loss (%) versus time were made.

### 2.3. Preparation of the Film-Forming Chlorhexidine Gluconate Sprays (CHG-FFS)

The FFS containing 0.5% *w*/*w* of CHG were prepared with three different film-forming polymers of S, L, and PVA together with the excipients (Table 3). Briefly, the film-forming agent was solubilized in a mixture of solvents and stirred overnight in a closed container. The aqueous stock solution of CHG was added slowly under constant stirring. The dissolved propylene glycol and menthol in a small amount of the solvent were added dropwise under constant stirring. The coloring and flavoring agents were sequentially added to the prepared solution. The remaining solvent mixture was used to make up the weight. To prevent the solvents from evaporating, the solution was immediately transferred to a spray container.

### 2.4. Evaluation of the CHG-FFS and CHG Films

#### 2.4.1. In Vitro Evaluation

The CHG-FFS was evaluated for the parameters concerning the appearance, pH, viscosity, weight of the FFS delivered upon each actuation, and the evaporation rate of the solvent. The pH of the CHG-FFS was assessed at 25 °C using a calibrated Mettler Toledo Electrode (Mettler Toledo GmbH, Zurich, Switzerland) with a nonaqueous probe in order to ensure the non-irritation of the formulations to the skin. The viscosity of the CHG-FFS was measured at 25 ± 1 °C using a Brookfield viscometer (digital viscometer model DV-II+, Stoughton, MA, USA) at spindle rotational speeds of 50 rpm. The weight of the FFS delivered upon each actuation was determined by accurate weighting to four decimal places by an analytical balance after actuation onto a clean Petri dish. The evaporation rate of each CHG-FFS was also evaluated through the examination of the changes in the weight over time. The graphs of the weight loss (%) versus time were plotted, as described above.

The cast films of the CHG-FFS were prepared to investigate the effect of the formulation on the properties of the films because the spray of the CHG-FFS was extremely thin, and it was not feasible to determine the physical and mechanical properties. The CHG film was produced by the solvent casting method, whereby the FFS was transferred onto a glass plate and dried in a hot air oven set at 37 °C with an indoor temperature of 34–35 °C for 6 h. The appearance of the dried films was examined visually, and films were maintained at ambient temperature until testing was required. A micrometer (Mitutoyo No. 7301, Kawasaki, Japan) was used to measure the thickness of the CHG film at three different locations on a single patch. A texture analyzer (TA.XT plus, Stable Micro Systems, Surrey, UK) with a 5 kg load cell equipped with a tensile grip holder was used to assess the mechanical properties of the CHG films in terms of tensile strength and Young’s modulus. The films were cut into a size of 20 mm × 55 mm. The speed was 2 mm/s, and the tensile strength and Young’s modulus were recorded. The surface morphology of the film was investigated by recording scanning electron microscopy (SEM, Tescan Mira 3, Brno–Kohoutovice, Czech Republic). The CHG films were characterized for their chemical structure using Fourier transform infrared spectrophotometry (FT-IR, iD7 ATR accessary for Nicolet^®^ iS5 FT-IR spectrophotometer, Thermo Scientific, Cambridgeshire, UK) with a wave number range of 400–4000 cm^−1^. The CHG powder was also measured as the control.

#### 2.4.2. In Vivo Evaluation

The in vivo study of the CHG-FFS, including the satisfaction of the dosage forms, time required for the film formation, film appearance, and adhesive test was conducted in eight healthy human volunteers comprising two men and six women with an age range between 20 and 22 years. The objective of the study was fully explained, and the human volunteers gave their written consent. This study was carried out under the approval of the RSU Ethics Review Broad, Rangsit University (RSU-ERB2022/036, date of approval: 8 April 2022). The CHG-FFS was actuated three times every 10 s on the back of the hand of the healthy human participants from a distance of 15 cm. The time required for the film formation, film appearance, satisfaction of the flavor, feeling of a cooling sensation, and sensation on the skin were recorded. The satisfaction of the flavor was graded as satisfaction (✓) and no satisfaction (✕). The cooling sensation was graded as low (+), medium (++), and high (+++). Aside from the pleasure of evaluating the film drying time, the time required for the film formation was observed by recording the time from when the FFS was sprayed over the back of the hand until the volunteers notified the researchers that the film had dried. The appearance of the film was then observed and graded on a score of 1–4 with 1—transparent and shining, 2—transparent but without a shine, 3—transparent but flaky, and 4—whitish film [16]. The sensation of the skin was also recorded as smoothness (✓) or stickiness (✕).

The adhesive test of the CHG-FFS investigated the adhesion upon the application of the FFS formulation. The CHG-FFS was sprayed onto the back of the hand on an area size of 14.5 cm^2^ and left for 5 min. Dry or wet swabs (soaked in distilled water) were performed four times on the films. Each swab was placed on the film for 5 s. CHG was extracted with distilled water from the swabs. The CHG content was analyzed by high-performance liquid chromatography analysis (HPLC) (Agilent Technologies Inc., Santa Clara, CA, USA). Luna 5u (Phenomenex, Torrance, CA, USA) C18 column (5 mm, 4.6 nm × 150 mm) were used. The mobile phase consisted of methanol/glacial acetic acid/ultrapure water (55/1/44, *v*/*v*). The flow rate was 0.5 mL/min, and the UV detection wavelength was 239 nm at 25 °C.

### 2.5. Antibacterial Activity of the CHG-FFS

#### 2.5.1. In Vitro Activity

The antiseptic activity was evaluated against *Staphylococcus aureus* ATCC 25923 (*S. aureus*). The agar diffusion method was used to determine the inhibition zone against *S. aureus*. *S. aureus* was incubated in Mueller–Hinton broth (MHB) at 37 °C for 18 h. The bacterial pellets were then dispersed in normal saline solution with the turbidity adjusted at 0.5 McFarland (10^8^ CFU/mL). The Mueller–Hinton agar (MHA) plates were spread by the bacterial suspension before being tested. Afterwards, the CHG-FFS was dropped into round shapes with 6 mm diameters and placed on the surface of the MHA plates. The diameter of the inhibition zone was measured after 18 h of incubation at 37 °C. The CHG solution and the negative control (disc without the sample) were also tested. The study was conducted in triplicate.

#### 2.5.2. In Vivo Activity

The in vivo antiseptic activity was also conducted on healthy human volunteers with no clinical signs of dermal abrasion, trauma, and infection. This evaluation was also performed under ethical approval (RSU Ethics Review Broad, Rangsit University, RSU-ERB2022/036, date of approval: 8 April 2022). The human volunteers were asked to pull their fingertips at a nearly horizontal angle for 2 s on the MHA plate. After that, the same fingertips were thrice sprayed with the CHG-FFS, allowed to dry for 2 min, and put again on the MHA plate. The MHA plates were incubated at 37 °C for 24 h before observing the bacterial colonies. The percentage of the bacterial reduction after the application of the CHG-FFS was calculated. The test of the other CHG-FFS formulation was then taken from the other hand. The 70% *v*/*v* of EtOH and available iodine (Betadine^®^) were also tested.

### 2.6. Indirect Cytotoxicity Evaluation

The cytotoxicity of the blank film and CHG film was assessed for normal human fibroblasts (NHFs). The NHF cultures were incubated at 37 °C in a humidified atmosphere of 95% confidence air and 5% CO_2_ in Dulbecco’s modified Eagle’s medium (DMEM) supplemented with 10% fetal bovine serum (FBS), 1% non-essential amino acid solution, and % penicillin-streptomycin solution. The blank film and CHG film were sterilized by UV radiation for 1 h before submerging in a serum-free medium (SFM) solution in an incubator for 24 h. A total of 10,000 NHF cells were seeded per well in 96-well plates in a volume of 200 μL. When the cultures had attained the confluence, the cells were treated with the extraction media containing various concentrations of the blank film and CHG film ranging from 0 to 2000 μg/mL and further incubated for 24 h. The control cells were added to the equivalent volume of fresh media. After the treatment, the cytotoxicity was examined by a 3-(4,5-dimethylthiazol-2-yl)-2,5-diphenyl-tetrazolium bromide (MTT) assay. The cells were incubated with MTT solution in a CO_2_ incubator for 1 h. The solution was then discarded, and DMSO was used to dissolve the formazan crystals. The cell viability was determined based on the absorbance at 550 nm. The non-treated control cells were arbitrarily designated as having 100% vitality.

### 2.7. In Vitro Scratch Test

The migration abilities of the NHF cell were also employed to test the wound healing effect using the scratch assay. The cells were seeded in a 6-well plate at a density of 1 × 10^6^ cells/mL in a volume of 1 mL. When the monolayer formed, a sterile pipette tip was used to make a linear scratch, which was subsequently washed with phosphate-buffered saline (PBS). After the scratch defect was made, the cells were exposed to the extraction media with the blank film and CHG film at the concentration of 1000 μg/mL. Non-treated control cells served as a control. Images were taken of the cells after the scratch defect was produced, after which the cells were immediately returned to the incubator at 37 °C. Subsequent images of the scratch defects were obtained at 6 h and 24 h. The photographs for each sample were then quantitatively examined by using image analysis software (JMicro Vision V.1.2.7, Geneva, Switzerland). The percent migration rate was obtained after measuring the distance of each scratch closure.

### 2.8. Statistical Analysis

All experimental measurements were collected at least triplicate. The data were expressed as the means ± standard deviation (SD). The statistical data were analyzed by Student’s *t*-test, and the significance level was set to *p* < 0.05.

## 3. Results and Discussion

### 3.1. Film-Forming System Formulation

**Figure 1:**
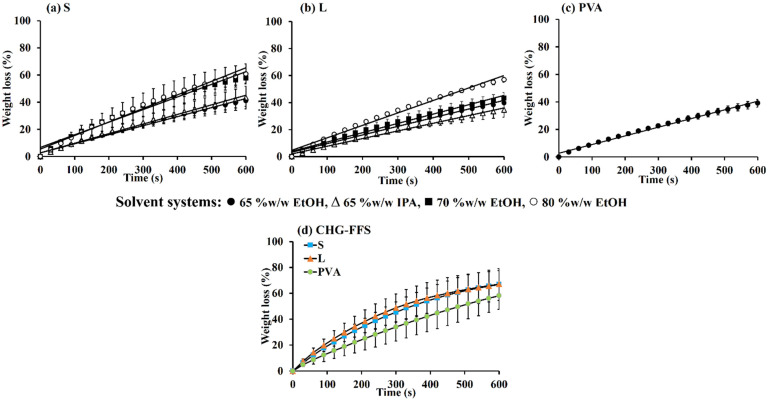
The percentage of the weight loss presented as the evaporation rates of the FFS at different types, and concentrations of the solvent systems at room temperature of 25–28 °C. The experiment was repeated three times, and the results were presented as the mean ± SD.

By varying the type of polymers, the film-forming polymer required to make a transparent film was determined (Table 1). Based on the visual observations regarding the film appearance, the water-soluble polymers (ALG, CMC, PVA, and PVP K30) and water insoluble polymers (E, L, S, and RS) were found in clear and transparent solutions with varying viscosities, which depended on the nature of the polymer. After the solvent in the solutions had evaporated, the dry film was identified. The PVA, L, and S films were found to be clear and transparent, while the rest were opaque or powdery (Table 1). As a result, PVA, L, and S were considered to determine the appropriate type and concentration of the solvent systems. To show the influence of the solvent systems on the appearance of the FFS and the rate of solvent evaporation, 65% *w*/*w* EtOH and IPA solutions were used as the solvents. Aqueous EtOH and IPA solutions have been widely employed as solvents in topical formulations in pharmaceuticals and cosmetics. Because of the solubility of the polymer in various solvents, S, L, and PVA obviously dissolved in 65% *w*/*w* of EtOH; however, only S and L dissolved in 65% *w*/*w* of IPA. The PVA was water soluble, but slightly soluble (95%) in the EtOH and insoluble in organic solvents [17]. In organic solvents, S and L were soluble [17]. Figure 1 illustrates the evaporation rate of the spray on the solutions by showing the percentages of the weight loss versus time by using slope scaling to represent the solvent evaporation rate (Table 2). The evaporation rate curve indicated a constant weight loss in the percentage. The theoretical maximum weight loss of the FFS was 98.5% for various types and concentrations of the polymers and solvent systems (Figure 1a–c). According to the room temperature of 25–28 °C throughout the experiment, the weight loss did not begin to saturate after 600 s. However, the solvent behavior in terms of weight loss and the evaporation rate could be sufficiently clarified during this period of time. When the evaporation rates of the EtOH and IPA were compared, the EtOH evaporated slightly faster (Table 2, Figure 1a,b). The rate of the weight loss was slightly increased when 65% *w*/*w* of EtOH was used. This was due to EtOH’s (47.07) lower molecular weight than that of IPA (60.10) [17]. Furthermore, the concentration level of the EtOH influenced the rate of evaporation. As the EtOH concentration was increased, the rate of solvent evaporation as well as the rate of the weight loss increased. However, because the CHG dissolved in water with no more than five parts of alcohol, and no more than three parts of acetone [7], the maximum EtOH concentration was thus up to 80% *w*/*w*. The solvent mixtures with the highest rate of solvent evaporation were selected for further formulation development based on the results. The PVA was made with 65% *w*/*w* of EtOH, while S and L were prepared with 80% *w*/*w* of EtOH.

### 3.2. Formulations and Physicochemical Properties

The CHG was prepared as FFS using three different film-forming polymers (S, L, and PVA) that dissolved in an aqueous ethanolic solution. For the topical skin disinfectant and antiseptic application, 0.5% *w*/*w* of CHG was used [7]. In addition, a mixture of CHG in an alcoholic solution was employed to improve the efficacy [7]. At concentrations of 60 to 95%, EtOH was bactericidal in aqueous mixtures [17]. The CHG was freely soluble in water (800 mg/L) [18] and dissolved in an aqueous solution containing no more than five parts of alcohol [7]; therefore, a preliminary analysis of CHG dissolved in 65 and 80% *w*/*w* of EtOH was carried out. The precipitation of the CHG was examined after allowing the solutions to stand at room temperature, and no precipitation was detected, indicating that the CHG and these solvent systems were compatible. In addition, these solvent systems were considered to be safe due to their reported use and general acceptability in pharmaceutical products. According to the results of the polymeric films, the PVA produced a flexible film, whereas S and L generated brittle films after being sprayed onto the skin. Consequently, the plasticizer was necessary for S and L, as it helped in the reduction in the glass transition temperature of the polymer. The film became more flexible as a result of this, which lowered cracking and flaking [19]. Transparent, non-flaky films having good flexibility of S and L were also obtained using the plasticizers; specifically, propylene glycol. Menthol was added to the formulations to provide the skin with a cooling sensation and to improve skin penetration [20]. Table 3 shows the constituent of the CHG-FFS.

Table 4 shows the characteristics of the CHG-FFS. All CHG-FFS formulations were a green, clear, uniform, and transparent solution with viscosity. The pH of the formulated FFS ranged from 5.59 to 5.92, which was the range of the human skin that was between 5.0 and 6.0 [21]. The viscosity of all formulations ranged from 8 to 11 cPs, which was a result of the difference in the kind and concentration of the polymers [22]. The importance of the viscosity was derived from the fact that it affected droplet spread. The spray would not be evenly spread throughout the target surface before evaporation if the viscosity was too high [23]. All FFSs were found to provide the optimum balance of spray ability and viscosity required for the spray to remain on the surface without dripping and interacting with it until drying. The weight of the FFS delivered upon each actuation ranged from 70.12 to 73.42 mg. If the spraying time was increased, the weight of the FFS also tended to rise. The percentage of the weight loss of the CHG-FFS as a function of time is shown in Figure 1d. The theoretical maximum weight losses of the CHG-FFS were 86.45, 86.45 and 97.95% for the S, L and PVA formulation, respectively. The results show that the initial evaporation rate was considerable, as the line sloped rapidly upward, but this was followed by a plateau as the weight loss slowed down. This phenomenon was not the same as the polymer solution in the ethanolic solution. Additionally, the CHG might affect the evaporation of the solvent systems. Furthermore, a number of other factors influenced the rate of evaporation, including the temperature, air movement, and humidity [24]. To compare the rate of the weight loss of each FFS, S and L were similar, but the PVA had a slightly lower rate due to the solvent system’s lower EtOH (Table 4).

**Figure 2:**
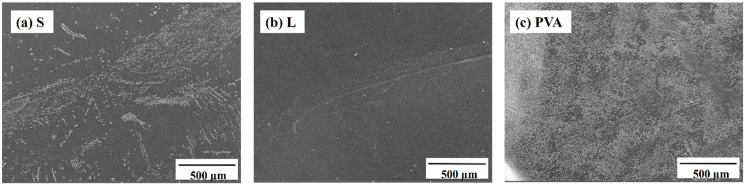
The surface morphology of the CHG film under the SEM.

The CHG-FFS formulations with various film-forming polymers formed as homogenous solid films and their physicochemical properties were examined (Table 4). All formulations produced a green, clear, homogeneous, and transparent film. Figure 2 also shows the surface morphology of the CHG films. The surface of the CHG films appeared to be relatively smooth, hence indicating that the CHG was well-incorporated into the films with small spherical particles on the surface that could be menthol particles generated during the solvent evaporation. The amount of solid mass added to the CHG-FFS formulations determined the CHG film thickness, which ranged from 0.035 to 0.077 mm. The CHG film from S and L had a two-fold higher solid mass than that obtained from the PVA (Table 3), consequently resulting in a two-fold thicker CHG film (Table 4). Because the FFS was intended to be used on wider regions of the skin, it was important to ensure that they would have sufficient flexibility and elasticity. A lack of these attributes would result in cracks and fissures interrupting the film as the patient moved. As a result, the stress–strain curves of the tensile tests could be used to measure the film’s mechanical properties. In addition, a plasticizer (PG) embedded in the S and L films resulted in a decrease in the tensile strength and an increase in the film’s elongation [25]. Plasticizers could reduce the rigidity of the polymer structures by reducing the number of binding sites between the polymer–polymer interactions [25]. The –OH group in the low-molecular weight PG inhibited the intermolecular hydrogen bonding of the S and L polymers, thus resulting in a weaker interaction between the polymer and PG [26]. In addition, the PVA film provided flexibility without the need for a plasticizer. Moreover, the PVA was shown to exhibit plastic behavior, thereby allowing the films to maintain the structural integrity and continuity under abnormal external stress [27]. Table 4 also shows the effect of the film composition on the film’s mechanical properties. The mechanical properties of the CHG films made from S, L, and PVA were similar, including the tensile strength and Young’s modulus.

**Figure 3:**
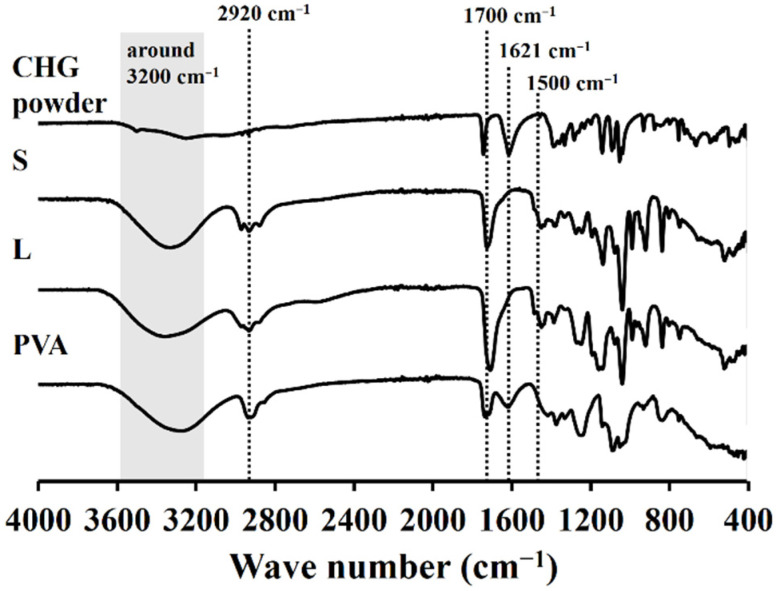
FTIR spectra of CHG powder and film.

Figure 3 shows the FT-IR spectra obtained for the CHG powder and film. The CHG characteristic peaks at 1500–1700 cm^−1^ were observed in the FT-IR spectra. At around 3200 cm^−1^, the FTIR spectra of the CHG showed a band associated with the N-H and –OH stretching. The vibrations of CH stretching were responsible for the signal at 2920 cm^−1^. The C=N stretching vibration had a peak at 1621 cm^−1^. These CHG absorption peaks corresponded with the report of Hosseini et al. [28]. The films exhibited the characteristic absorption peaks of the CHG, indicating that the CHG molecules had been successfully loaded into the polymeric network of films.

### 3.3. In Vivo Evaluation

Table 5 shows the in vivo study of the CHG-FFS, including the appearance of the film and satisfaction of the dosage forms. After actuation, all CHG-FFS formulations formed the film on the skin of healthy human volunteers. The rate of film formation was determined by the evaporation of the solvents, leaving a residue of film with an effective CHG concentration for antiseptic activity. The CHG-FFS of S, L, and PVA transformed to give shiny and transparent films for most human volunteers. One individual was responsible for providing a white film for S and L. This outcome occurred because of the effect on the surrounding environment, including the body temperature of the human. When the solvent evaporated quickly, the polymer-containing droplets dried before reaching the skin or failed to spread over the skin’s surface, resulting in an orange peel effect or whitish film [24]. However, the CHG film had good dermal adhesion, flexibility of the film, and film formation in a shorter duration of time of 54.25 ± 5.15, 50.74 ± 7.82, and 47.20 ± 3.51 s for the S, L and PVA film, respectively. These results demonstrate that the CHG-FFS showed good spray properties, clear transparent film after application, and drying time of less than a minute. Because of the menthol in the formulation, a cooling effect was reported following the application of the CHG-FFS ranging from medium to high. Overall, the flavor stratification was successful. The flavor of the formulation was disliked by only one person. During the application of the CHG-FFS, more than half of the participants reported a feeling of smoothness on their skin.

**Figure 4:**
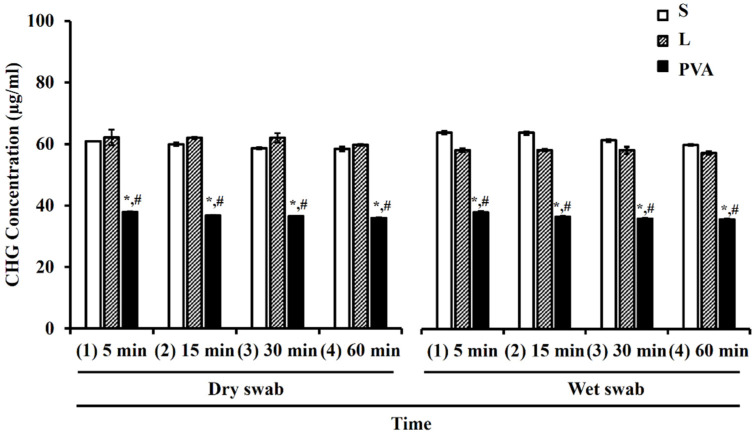
The concentration of the CHG loss from the skin of the healthy volunteers depended on the number of dry or wet swabs after spraying the CHG-FFS. The experiment was repeated three times, and the results were presented as the mean ± SD. * and # statistically significant (*p* < 0.05) with S and L, respectively.

The adhesive test of the CHG-FFS was carried out to confirm that the adhesion improved after the FFS formulation was applied. As the solvent evaporated, the S, L, and PVA in the FFS created a film that prevented the crystallization of the CHG. As a result, the FFS might significantly improve the CHG’s adhesion and prolong the duration of the CHG’s exposure on the skin, consequently boosting the CHG’s antibacterial activity and patient’s convenience by lowering the application frequency [29]. Figure 4 shows the results of adhesion test of three formulations depending on the number of swabs. The concentration of the CHG loss from the skin of healthy volunteers depended on the formulation and the number of dry or wet swabs. Overall, the CHG-FFS of the PVA exhibited much higher adhesion ability than that of S and L. After the dry or wet swab, the CHG film of the S and L showed a high CHG loss (60 µg/mL). On the other hand, the CHG film of PVA exhibited significantly lower CHG loss (40 µg/mL, *p* < 0.05). The concentration of the CHG loss was constant, as the number of swabs increased as a function of time. Furthermore, no significant differences in CHG loss were found between the dry and wet swabs. These might be due to the short duration of each swab. After a few seconds, the CHG was slightly diffused from the film base to the dry and wet materials. In addition, S and L were soluble in neutral to weakly alkaline conditions (pH 6–7), whereas PVA required water at a temperature of 90 °C to dissolve [17]. These polymeric properties demonstrated that the CHG was not easily lost under wet conditions. Additionally, the dry and wet swabs illustrated how CHG film performed on the skin in dry conditions, and when it came into contact with water or sweat, respectively [2]. These results reveal that after spraying the FFS formulation, the adhesion improved significantly, and the CHG was lost from the PVA film at a lower rate in both the dry and wet skin conditions.

### 3.4. Antibacterial Activity

**Figure 5:**
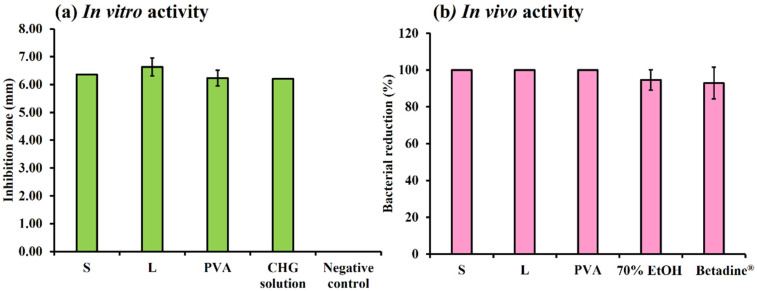
(**a**) In vitro and (**b**) in vivo antiseptic activity of the CHG-FFS. The experiments were repeated three times, and the results were presented as the mean ± SD.

**Figure 6:**
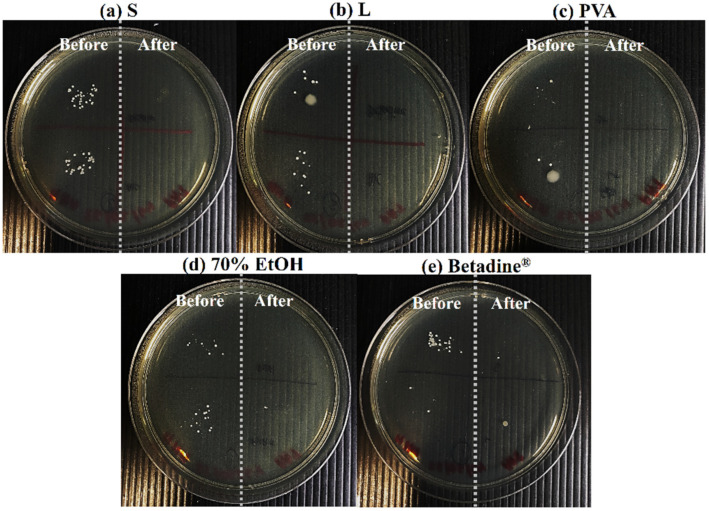
MHA plate with bacteria before and after the application of the CHG-FFS.

In patients of all ages, *S. aureus* generally causes skin infections in the epidermis and upper dermis structures [30]. It also colonizes the skin and mucosa surfaces frequently. Topical antibiotics and antiseptics are frequently used to prevent *S. aureus* colonization. The results of disk diffusion are shown in Figure 5a, representing the diameter of the inhibition zone, including the diameter of the paper disk. The negative control, the disc without the sample, could not inhibit the growth for *S. aureus*, thus demonstrating that the experimental condition was suitable for the antibacterial activities test. All the CHG-FFS formulations inhibited the growth of *S. aureus*, which were significantly greater than the negative controls. *S. aureus* was susceptible to CHG released from the FFS formulation. However, the antibacterial activity of the CHG-FFS and CHG solution was non-significantly different against *S. aureus*. The inhibition zone of the FFS formulation with the various polymers was similar, which indicated that the type and amount of the film-forming polymers in the FFS did not affect the antibacterial activity of the CHG. The CHG was well-incorporated into the FFS and retained its antibacterial action. The antibacterial activity did not alter when the CHG was formulated into the FFS formulation. The in vivo activity was also examined in healthy human volunteers to confirm the results in vitro. The number of bacteria on the participants’ fingers decreased by almost 100% after they were sprayed with CHG-FFS (Figure 5b). The bacterial reduction with the different polymers was likewise similar to confirming the in vitro investigation that film-forming polymers in the FFS had no effect on the CHG’s antibacterial activity. Furthermore, the antibacterial efficiency of the CHG-FFS was similar to those of standard 70% *v*/*v* EtOH and commercially available iodine (Betadine^®^). Figure 6 shows the images of the bacterial colonies before and after the CHG-FFS, 70% EtOH, and Betadine^®^ application. It was demonstrated that applying the CHG-FFS reduced the number of bacteria on the skin by acting as an antiseptic. CHG had a wide range of effects, including Gram-positive and Gram-negative bacteria, fungi, enveloped viruses, and protozoa [31]. The positively charged CHG molecule bonded to the negatively charged lipid bacterial cell surface, weakening the cell membrane integrity and causing cytoplasm leakage and protein and nucleic acid precipitation [8]. The breakdown of cell membrane components and dehydrogenase activation generated by a low concentration of CHG (0.5% *w*/*w*) comprised bacteriostatic activity [31].

### 3.5. Cytotoxicity and Wound Healing Evaluation

**Figure 7:**
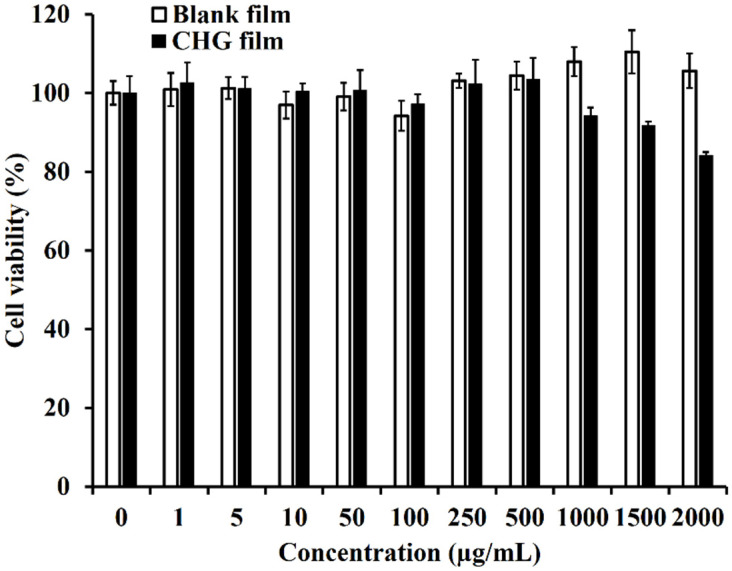
Indirect cytotoxicity of the films to the NHF cells. The cell viability was determined by the MTT assay. The experiment was repeated five times, and the results were presented as the mean ± SD.

CHG-FFS formulated with PVA was considered as a promising formulation to study the cytotoxicity and wound healing effect according to the satisfactory physical properties of the solution to provide a good film both in vitro and in vivo. Topical antibacterial dosage forms should, in general, not only improve antibacterial efficacy but also be low in cytotoxicity to skin cells. The NHF cells were used to test the cytotoxicity of all films formulated with PVA for 24 h. Figure 7 exhibits the cell viability of the various film concentrations. When the cells were incubated with the blank film at the tested doses (0–2000 µg/mL), there was no significant reduction in cell viability when compared to the untreated cells because PVA was biocompatible, non-toxic, and non-irritant. These results reveal that the blank film had no effect on the cell viability during the 24 h incubation period, hence demonstrating excellent biocompatibility. The cell viability of the various concentrations of the CHG film is also presented in Figure 7. The cell viability was not significantly different from the non-treated cells and respective concentration of the blank film when the NHF cells were treated with the CHG film at ≤500 µg/mL (*p* > 0.5). As the CHG film concentration was increased from 1000 to 2000 µg/mL, the NHF viability was significantly reduced when compared to non-treated cells and the blank film concentration. However, the cell viability was more than 80%, indicating that the CHG film had less cytotoxicity and was safe on the skin at concentrations of 0–2000 µg/mL within a 24 h incubation period. Because the solvents quickly evaporated after spraying, a thin film formed in a short time, causing low toxicity in human skin cells in vitro.

**Figure 8:**
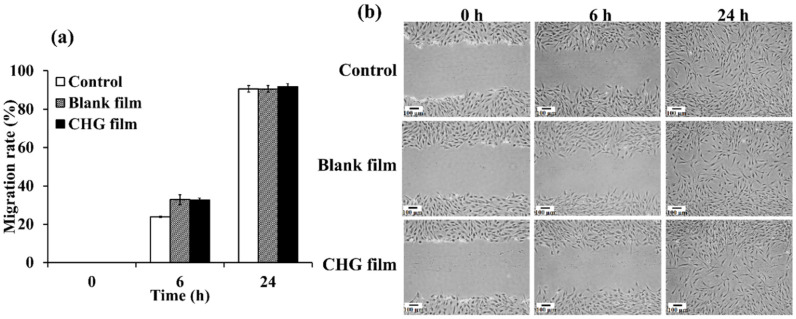
Evaluation of the cell migration in the in vitro scratch assay; (**a**) time-dependent effects of the blank and CHG films on the scratch closure rate. (**b**) Photographs of the NHF cells treated with the control, blank and CHG films at 0 h, 6 h, and 24 h after incubation.

The blank and CHG films were evaluated for the rate of migration of the NHF cells. The in vitro scratch assay was examined at 0, 6 and 24 h to mimic the migration of the cells [32]. The proliferation and migration of fibroblasts were observed in the scratch assay, which covered the second phase of wound healing [33]. In comparison to the control, the presence of the blank and CHG films had no effect on cell growth and migration, and the film did not promote wound healing. The cell migration rate of the blank and CHG films were similar to the control at 6 h and 24 h (Figure 8a). This could be attributed to the fact that the films did not improve the growth factors or wound healing related with cytokines for cell proliferation, adhesion, migration, differentiation, extracellular matrix deposition, and wound healing regulation [34]. As shown in Figure 8b, all films, including the control, showed a closure of the gap at 24 h. However, the length between the scratch marks in the CHG films was slightly more loosely closed than the control and blank film. The effect of CHG on the cells was reasonable. Nevertheless, the FFS forming the films might reduce the cytotoxicity, resulting in less inhibition of the cell migration and wound healing. The literature reported that the solution of CHG of ≥0.02% had a toxic effect and scratch defects on the fibroblasts, myoblasts, and osteoblasts, respectively [14,35]. These results confirm that CHG formulated to the FFS and film delivery did not interfere with the cell proliferation and migration in the wound healing process.

## 4. Conclusions

The CHG-FFSs were successfully prepared using S, L and PVA as the film-forming polymers in the aqueous ethanolic solution. A transparent homogenous spray, clear, and flexible film after actuation was obtained. PVA was found to be the optimal film-forming polymer for promoting the CHG adhesion on the skin. This generated the fast thin film formation on the skin with dosage form satisfaction. The formulation inhibited the growth of *S. aureus* in vitro and provided an antiseptic activity in vivo. Moreover, the CHG film was non-toxic toward the human skin cells in the cell culture without any interference in the wound healing process. Thus, CHG-FFS may be a potential candidate for topical antiseptic application.

## Figures and Tables

**Table 1:** Physical appearance of FFS and polymeric films.

Film-Forming Polymers	Solvent	FFS		Film
		Appearance	Viscosity *	
ALG	Purified water	Transparent solution	Medium	Opaque film
CMC	Purified water	Transparent solution	High	Opaque film
PVA	Purified water	Transparent solution	Low	Transparent and flexible film
PVP K30	Purified water	Transparent solution	Low	Sticky film
E	95% *v*/*v* EtOH	Transparent solution	Very low	Powder-like film
L	95% *v*/*v* EtOH	Transparent solution	Very low	Transparent and brittle film
S	95% *v*/*v* EtOH	Transparent solution	Very low	Transparent and brittle film
RS	95% *v*/*v* EtOH	Transparent solution	Very low	Powder-like film

* Viscosity: very low ≤ 12 cPs, low = 12–35 cPs, medium = 35–80 cPs, and high = 80–100 cPs.

**Table 2:** Physical appearance and rate of the weight loss of the FFS prepared from the various types and concentrations of the solvent systems.

Film-Forming Polymers	Solvent	Physical Appearance *	Rate of Weight Loss (%/s)
S	65% *w*/*w* IPA	Transparent solution; very low viscosity	0.0677 ± 0.0103
	65% *w*/*w* EtOH	Transparent solution; very low viscosity	0.0708 ± 0.0112
	70% *w*/*w* EtOH	Transparent solution; very low viscosity	0.0930 ± 0.0105
	80% *w*/*w* EtOH	Transparent solution; very low viscosity	0.0997 ± 0.0126
L	65% *w*/*w* IPA	Transparent solution; very low viscosity	0.0568 ± 0.0088
	65% *w*/*w* EtOH	Transparent solution; very low viscosity	0.0642 ± 0.0126
	70% *w*/*w* EtOH	Transparent solution; very low viscosity	0.0690 ± 0.0138
	80% *w*/*w* EtOH	Transparent solution; very low viscosity	0.0922 ± 0.0127
PVA	65% *w*/*w* IPA	Precipitation	N.D.
	65% *w*/*w* EtOH	Transparent solution; low viscosity	0.0630 ± 0.0091
	70% *w*/*w* EtOH	Precipitation	N.D.
	80% *w*/*w* EtOH	Precipitation	N.D.

* Viscosity: very low ≤ 12 cPs, low = 12–35 cPs, medium = 35–80 cPs, and high = 80–100 cPs. N.D. = Not detected.

**Table 3:** Constituent of CHG-FFS.

Ingredient (% *w*/*w*)	Formulation			Functions
	S	L	PVA	
CHG	0.5	0.5	0.5	Antiseptic agent
PVA	-	-	1.5	Film-forming agent
S	3	-	-	Film-forming agent
L	-	3	-	Film-forming agent
Propylene glycol	10	10	-	Plasticizer
Menthol	0.05	0.05	0.05	Cooling agent
Tartrazine	qs.	qs.	qs.	Coloring agent
Brilliant Blue FCF	qs.	qs.	qs.	Coloring agent
Corn mint oil	qs.	qs.	qs.	Flavoring agent
Rosemarry oil	qs.	qs.	qs.	Flavoring agent
EtOH	80	80	65	Solvent
Purified water qs. to	100	100	100	Solvent

**Table 4:** In vitro evaluation of CHG-FFS and CHG film.

Dosage Forms	Evaluation	Formulation		
		S	L	PVA
CHG-FFS	Appearance	Green/transparent	Green/transparent	Green/transparent
	pH	5.82 ± 0.16	5.59 ± 0.03	5.92 ± 0.04
	Viscosity (cPs)	8.94 ± 0.43	10.67 ± 0.34	8.41 ± 0.69
	Weight of actuation (mg)	70.12 ± 11.17	73.42 ± 26.23	71.49 ± 21.65
	Rate of weight loss (%/s)	0.1086 ± 0.0131	0.1062 ± 0.0212	0.0951 ± 0.0181
CHG film	Appearance	Green/transparent	Green/transparent	Green/transparent
	Thickness (mm)	0.0750 ± 0.0084	0.0767 ± 0.0197	0.0350 ± 0.0138
	Tensile strength (N)	8.58 ± 1.71	8.05 ± 1.44	8.92 ± 2.35
	Young’s modulus (Pa)	0.285 ± 0.045	0.299 ± 0.064	0.382 ± 0.065

**Table 5:** In vivo evaluation of CHG-FFS.

Volunteer	Formulation											
	S				L				PVA			
	Appearance ^a^	Flavor ^b^	Cooling ^c^	Skin Feel ^d^	Appearance ^a^	Flavor ^b^	Cooling ^c^	Skin Feel ^d^	Appearance ^a^	Flavor ^b^	Cooling ^c^	Skin Feel ^d^
1	1	✓	+++	✓	1	✓	+++	✓	1	✓	+++	✕
2	1	✓	+++	✓	1	✓	+++	✓	1	✓	+++	✓
3	4	✓	+++	✕	4	✓	+++	✕	1	✓	+++	✓
4	1	✕	++	✓	1	✕	++	✕	1	✕	++	✕
5	1	✓	++	✕	1	✓	++	✕	1	✓	++	✕
6	1	✓	+++	✓	1	✓	+++	✓	1	✓	+++	✓
7	1	✓	++	✕	1	✓	++	✕	1	✓	++	✕
8	1	✓	+++	✓	1	✓	+++	✓	1	✓	+++	✓

^a^ (1) transparent and shining, (2) transparent but without a shine, (3) transparent but flaky and (4) whitish film; ^b^ (✓) satisfaction and (✕) no satisfaction; ^c^ (+) low, (++) medium and (+++) high; ^d^ (✓) smoothness and (✕) stickiness.

## Data Availability

The data that support the findings of this study are available from the corresponding authors, upon reasonable request.
